# Publicly available machine learning models for identifying opioid misuse from the clinical notes of hospitalized patients

**DOI:** 10.1186/s12911-020-1099-y

**Published:** 2020-04-29

**Authors:** Brihat Sharma, Dmitriy Dligach, Kristin Swope, Elizabeth Salisbury-Afshar, Niranjan S. Karnik, Cara Joyce, Majid Afshar

**Affiliations:** 1 Department of Computer Science, Loyola University Chicago, Chicago, IL USA; 2 Center for Health Outcomes and Informatics Research, Loyola University Chicago, 2160 S. First Avenue, Maywood, IL 60156 USA; 3 Stritch School of Medicine, Loyola University Chicago, Maywood, IL USA; 4 Center for Multi-System Solutions to the Opioid Epidemic, American Institute for Research, Chicago, IL USA; 5 Department of Psychiatry, Rush University Medical Center, Chicago, IL USA; 6 Department of Health Informatics and Data Science, Loyola University Chicago, Maywood, IL USA; 7 Department of Medicine, Loyola University Medical Center, Maywood, IL USA

**Keywords:** Opioid misuse, Heroin, Opioid use disorder, Natural language processing, Machine learning, Computable phenotype

## Abstract

**Background:**

Automated de-identification methods for removing protected health information (PHI) from the source notes of the electronic health record (EHR) rely on building systems to recognize mentions of PHI in text, but they remain inadequate at ensuring perfect PHI removal. As an alternative to relying on de-identification systems, we propose the following solutions: (1) Mapping the corpus of documents to standardized medical vocabulary (concept unique identifier [CUI] codes mapped from the Unified Medical Language System) thus eliminating PHI as inputs to a machine learning model; and (2) training character-based machine learning models that obviate the need for a dictionary containing input words/n-grams. We aim to test the performance of models with and without PHI in a use-case for an opioid misuse classifier.

**Methods:**

An observational cohort sampled from adult hospital inpatient encounters at a health system between 2007 and 2017. A case-control stratified sampling (*n* = 1000) was performed to build an annotated dataset for a reference standard of cases and non-cases of opioid misuse. Models for training and testing included CUI codes, character-based, and n-gram features. Models applied were machine learning with neural network and logistic regression as well as expert consensus with a rule-based model for opioid misuse. The area under the receiver operating characteristic curves (AUROC) were compared between models for discrimination. The Hosmer-Lemeshow test and visual plots measured model fit and calibration.

**Results:**

Machine learning models with CUI codes performed similarly to n-gram models with PHI. The top performing models with AUROCs > 0.90 included CUI codes as inputs to a convolutional neural network, max pooling network, and logistic regression model. The top calibrated models with the best model fit were the CUI-based convolutional neural network and max pooling network. The top weighted CUI codes in logistic regression has the related terms ‘Heroin’ and ‘Victim of abuse’.

**Conclusions:**

We demonstrate good test characteristics for an opioid misuse computable phenotype that is void of any PHI and performs similarly to models that use PHI. Herein we share a PHI-free, trained opioid misuse classifier for other researchers and health systems to use and benchmark to overcome privacy and security concerns.

## Background

Clinical notes from the EHR are promising for modeling prediction tasks in healthcare but they contain protected health information (PHI) and require legal and regulatory approvals to share across hospitals for implementation. The majority of published models that use machine learning for text classification are word-based classifiers that internally store a vocabulary of input features (e.g. word n-grams) [1–4]. Major challenges remain in privacy and security of PHI-laden models for sharing, and it prevents the deployment of NLP models at other hospitals.

Training NLP models in a way that prevents PHI leakage requires a prior step to manually or automatically scrub notes, which can be a laborious process and may not occur at all hospitals. These steps include employing software previously trained to identify and remove individual mentions of PHI [5–8]. Most software were trained on specific types of clinical documents, and many used pattern matching with rules and dictionaries and, more recently, machine learning. These systems do not have perfect accuracy so training machine learning algorithms with features derived from these systems may still lead to PHI embedded in the machine learning model. There remains a paucity of evidence examining approaches that can process and train an entire corpus of text documents in a PHI-free manner.

In this study, we experiment with the following solutions using feature engineering to provide PHI-free models for text classification: (1) using raw text converted into standardized medical vocabulary (concept unique identifiers [CUIs] mapped from the Unified Medical Language System); and (2) character-based models. CUI-based classifiers are PHI-free because their input features are concepts mapped to the Unified Medical Language System as standardized codes (e.g. ‘heroin’ is mapped to CUI code ‘C0011892’) that may be deployed across hospitals without concern for PHI leakage. A character-based model uses characters rather than words or n-grams as basic units of input; a classifier’s task in this case is to identify salient character sequences that are useful for the prediction task. Like CUI-based models, character-based models are PHI-free since their vocabulary consists of individual unique characters observed in the corpus of text.

Opioid misuse is a behavioral condition that represents a heterogeneous pattern of use rendering it complex to identify from the EHR and is an ideal use-case to examine our PHI-free approach. Patients with opioid misuse represent a vulnerable population so removing PHI for publicly available models is a priority. Opioid misuse is taking an opioid for reasons other than prescribed or as an illicit drug [9, 10]. Traditionally, diagnostic billing codes or rule-based models for opioid misuse have been used by health systems for health surveillance and monitoring of outcomes [11]. However, International Classification of Diseases-9 or-10 codes (ICD 9/10) are typically constrained by poor sensitivity/recall with a high false negative rate [12, 13]. Computable phenotypes that use supervised machine learning may learn the complexities of these behavioral conditions from the clinical notes to predict cases of opioid misuse.

We aim to compare the performance of multiple text classification approaches, including both PHI-laden and PHI-free, for an opioid misuse computable phenotype at a large, tertiary health system using routinely available EHR notes of hospitalized patients. A variety of machine learning models, including multiple neural network architectures that can contain hidden layers with PHI will be examined against our PHI-free approaches. We hypothesize that it is possible to build a PHI-free model for text classification of opioid misuse without sacrificing performance.

## Methods

### Population and setting

Loyola University Medical Center (LUMC) is a 559-bed hospital and tertiary academic center including a burn and Level 1 trauma center serving Chicago and its western suburbs. LUMC has maintained Epic (Epic Systems Corporation, Verona, Wisconsin) as its EHR vendor since 2003 and includes a Microsoft SQL server-based clinical data warehouse that has been available for research since 2007. The hospital cohort is comprised of 161,520 adult inpatient encounters (≥18 years of age) between January 1, 2007 and September 30, 2017. Patients with only outpatient encounters were excluded.

### Sampling of hospital cohort to build reference dataset for opioid misuse computable phenotype

The National Survey on Drug Use and Health (NSDUH) and the National Institute of Drug Abuse (NIDA) define opioid misuse as individuals taking an opioid for reasons other than prescribed or as an illicit drug [9, 10]. Opioid misuse is a behavioral condition that represents a heterogeneous pattern of use ranging from nonmedical prescription drugs to injection illicit drug use rendering it complex to identify from the EHR. To train and test our computable phenotype for opioid misuse, a random case-control sampling of 1000 patients was annotated from the hospital cohort during the study period for chart review. The study cohort for annotation included an oversampling of hospitalizations that had an ICD-9/10 codes for opioid-related hospitalizations or a positive urine drug screen for an opioid drug [14]. Additional sampling of at-risk patients included those who had ICD codes for chronic pain, naloxone (reversal drug for opioid overdose) order and administration, or a physician order for a urine drug screen. Age- and sex-matched controls without any of the above criteria were included as potential controls. A trained annotator (KS) performed review of each patient record to provide a final annotation for the likelihood of opioid misuse on a Likert scale (1–5). The annotator met an inter-rater reliability of Cohen’s kappa coefficient ≥ 0.75 with a critical care physician and substance use researcher (MA and ESA) before independent review was continued.

The Likert scale included definite, highly probable, probable, definitely not, and uncertain for determining opioid misuse in accordance with NIDA and NSDUH definitions. Probable cases required any one of the following: (1) history of opioid misuse evident in the clinical notes but no current documentation for the encounter; (2) provider mention of aberrant drug behavior; (3) evidence of other drug misuse (except alcohol) in addition to prescription opioid use. Highly probable cases were classified by more than one of the probable case criteria, or provider mention of opioid dependence plus suspicion of misuse in the clinical notes. Definite cases were classified as the patient self-reporting opioid misuse to a provider or documentation by provider of patient misusing an opioid. For the classification task of the computable phenotype, patients were categorized as exhibiting opioid misuse if they met probable, highly probably, or definite criteria – these were aligned with the definitions by NSDUH and NIDA. The remainder of cases of definitely not or uncertain were categorized as no opioid misuse. Only 1.9% (*n* = 19) of cases were classified as uncertain. The final reference dataset was comprised of 33.7% (*n* = 337) cases of opioid misuse.

### Rule-based opioid misuse computable phenotype for comparison to machine learning models

First, a simpler baseline rule-based model was built from structured data for comparison to machine learning models. The rules were developed by substance use specialists including an addiction specialist (ESA) and psychiatrist (NK), and in accordance with the NSDUH and NIDA definition for opioid misuse [9, 10]. The rule-based criteria for opioid misuse were met if any of the following structured data elements qualified from the EHR: (1) positive urine drug screen for an opioid with co-substance use with any of the following: an illicit drug (phencyclidine or cocaine), a benzodiazepine that was not self-reported by the patient as a prescribed medication, or an amphetamine that was not self-reported by the patient as a prescribed medication; (2) positive urine drug screen for an opioid only but not self-reported by the patient as a prescribed medication; (3) ICD-9/10 codes for opioid poisoning or intoxication [14].

### Machine learning models: PHI-free and PHI-laden

We experimented with several classes of models to learn the relationship between the input text and the classification task (opioid misuse vs. no opioid misuse) including linear classifiers and several neural network architectures. Encounter-level analysis (*n* = 1000) was performed by incorporating all clinical documents from each patient hospitalization for training the machine learning models.

PHI-laden models: We utilized n-gram classifiers mainly for comparison to PHI-free models (CUIs and character-based). A logistic regression model and convolutional neural network were trained with n-grams. The n-gram features were examined as unigrams, bigrams, and the combination of the two for the linear model and a 300-dimension word embedding for the neural network architectures. We also investigated the possibility of removing PHI from the n-gram models by training a token-based L1-regularized logistic regression model. Unlike L2-regularized models that often contain thousands of features, the sparsity of L1-regularized model allows manual examination of the features with non-zero weights to allow for manual removal of PHI-containing features post-hoc.

**Fig. 1:**
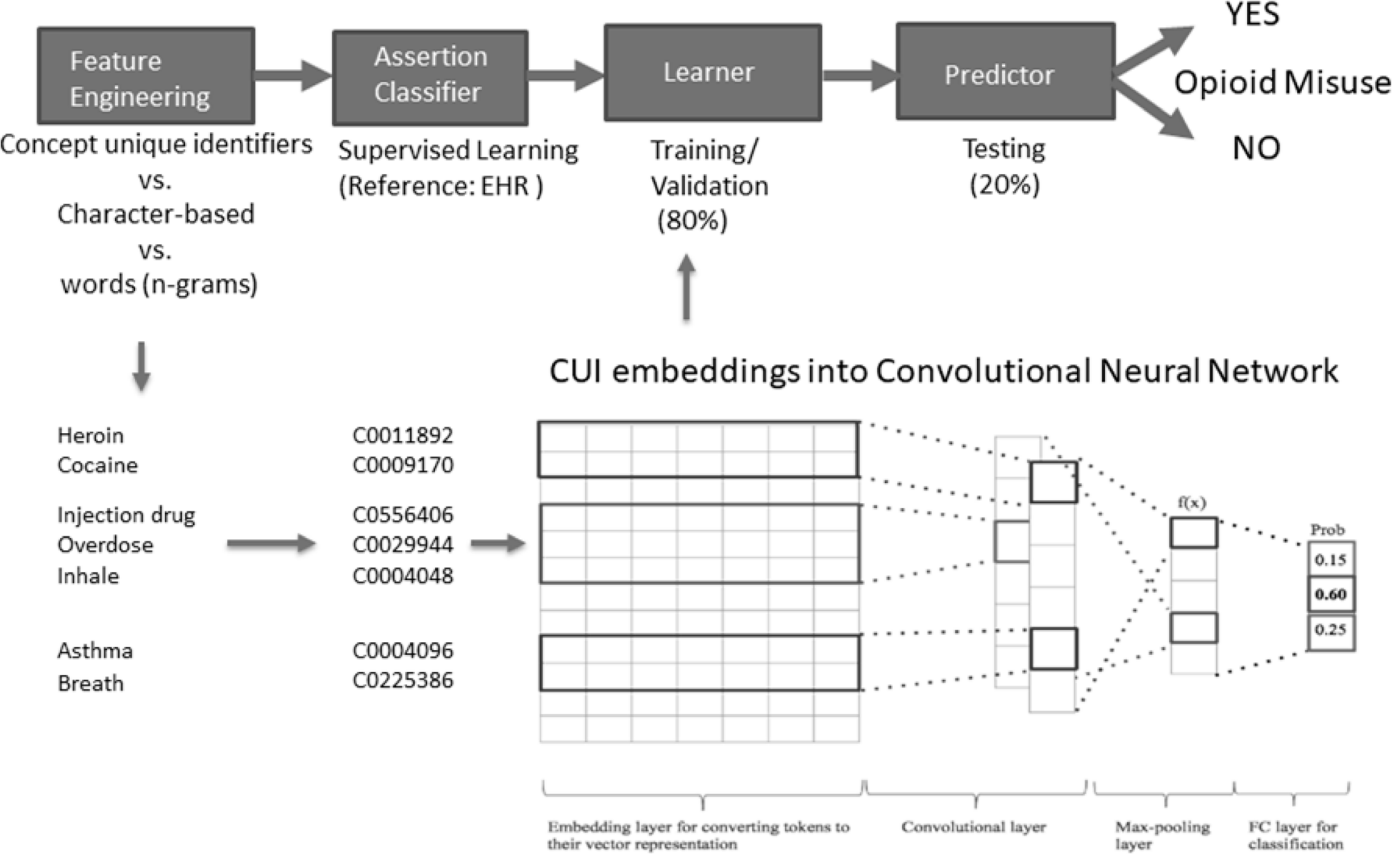
PHI-free and PHI-laden inputs to a machine learning model with an example of a convolutional neural network using an embedding with Concept Unique Identifiers (CUIs)

PHI-free models: We explored both CUI and character-based CNN models. Linguistic processing of the clinical documents into CUI codes was performed in clinical Text and Knowledge Extraction System (http://ctakes.apache.org) [15]. Named entity mentions in the raw text were mapped to the Unified Medical Language System (UMLS), which includes over 2 million concepts from nearly 9 million distinct names that are merged into the National Library of Medicine’s Metathesaurus. The spans of the UMLS named entity mentions (diseases, symptoms, anatomy, and procedures) were mapped from the raw notes and organized into Concept Unique Identifiers (CUIs). The free text in clinical documents from the EHR are matched to a dictionary of concepts, by default SNOMED CT and RxNORM, and tagged with concept codes from the original dictionary, and a CUI code from the UMLS Metathesaurus. This dictionary lookup puts text into a coded format that completely de-identifies to structured data as SNOMED CT and RxNORM CUI codes. For instance, the named entity mention for *‘heroin abuse’* has the CUI code ‘C0600241’. Each named entity mention was also analyzed to determine its negation status (e.g. ‘no heroin abuse’). The original cTAKES publication demonstrates that concept mapping and negation status had F1 scores of 0.957 and 0.943 on certain tasks, respectively [15]. Figure 1 represents an example of a CNN-based model for an opioid misuse computable phenotype.

The dataset was split into training (60%), validation (20%), and test (20%) data. The training set was used for training models, the validation set for parameter tuning, and the test set for the final performance evaluation. Term frequency-inverse document frequency (tf-idf) was applied to normalize the n-gram and CUI codes prior to being fed into the logistic regression models. The neural network architectures considered included: (1) a convolutional neural network (CNN) [13]; (2) a deep averaging network ( [16]; (3) a max pooling network [17]; and (4) combination of deep averaging and max pooling network. The CUI-based CNN includes 1024 filters of size 1. The filter of size one is used because the ordering of CUI codes in clinical texts is typically not meaningful and does not warrant larger filter sizes. A deep averaging network is a simple neural architecture that accepts word embeddings as inputs and averages them. The averaged values are then fed into a classification layer (sigmoid or softmax). The deep averaging network was first introduced in 2015 [17] and later re-introduced in 2016 [18]. This architecture can often be as effective as more complex models such as CNN or recurrent neural network [19]. A max pooling network picks the maximum value across each embedding dimension. The deep averaging and max pooling architectures project the resulting patient representation onto a dense layer to capture the interrelations between semantic dimensions. The dense layer is followed by a sigmoid output. All neural network models included a dropout layer for regularization and are trained using the Adam optimizer with binary cross-entropy loss. Random search was used for tuning the neural network hyperparameters in the validation cohort [20].

### Model evaluation and comparisons

The models were tuned to the highest area under the receiver operating characteristic curve (AUROC). Both neural networks and logistic regression models were examined across multiple hyperparameters (Table 1). Once the hyperparameters for each model were finalized, we combined the validation and training sets and evaluated the model in the independent test set.

**Table 1:** Machine learning models with hyperparameters

Model	Hyper-parameters
Logistic Regression-CUIs	C = 1, penalty = L1, class_weight = balanced
Logistic Regression-Words	C = 1, penalty = L1, class_weight = balanced
Convolutional Neural Network-CUIs	Filters = 1024, Filter Size = 1, Dropout = 0.5, Units = 1024, Learning Rate = 0.0001
Convolutional Neural Network-Words	Filters = 1024, Filter Size = 3, Dropout = 0.25, Units = 128, Learning Rate = 0.0001
Convolutional Neural Network-Character	Filters = 1024, Filter Size = 11, Dropout = 0.25, Units = 1024, Learning Rate = 0.0001
Deep Averaging Network-CUIs	Dropout = 0.25, Units in layer 1 = 2048, Units in layer 2 = 512, Learning Rate = 0.001
Deep Averaging Network-Words	Dropout = 0.75, Units = 128, Learning Rate = 0.001
Max Pooling Network-CUIs	Dropout = 0.5, Units = 128, Learning Rate = 0.001
Max Pooling Network-Words	Dropout = 0.5, Units = 128, Learning Rate = 0.001
Deep Averaging + Max Pooling Network-CUIs	Dropout = 0.5, Units = 1024, Learning Rate = 0.001
Deep Averaging + Max Pooling Network-Words	Dropout = 0.25, Units = 512, Learning Rate = 0.001

Logistic regression’s C value is inverse of regularization strength, and penalty term that penalizes the loss function using different regularization techniques. Optimizer Adam is selected for all the neural networks. Units are the number of neurons in the dense layer of the neural network

Discrimination of the prediction models was evaluated using the AUROC. Goodness-of-fit was formally assessed by the Hosmer-Lemeshow test and verified visually with calibration plots. Test characteristics (sensitivity/recall, specificity, negative predictive value (NPV), precision/positive predictive value (PPV) and macro F1 score) were provided to compare between classifiers. The DeLong et al. method was used to compare the AUROC between models [21]. For the logistic regression model, the beta-coefficients of the selected features were listed to examine face validity. Analysis was performed using Python Version 3.6.5 (Python Software Foundation) and RStudio Version 1.1.463 (RStudio Team, Boston, MA). The study was approved by the Loyola University Chicago Institutional Review Board (LU #209950). Need for consent was waived by the IRB and deemed unnecessary according to national regulations. The PHI-free models and the relevant code are publicly available in our GitHub repository (https://github.com/AfsharJoyceInfoLab/OpioidNLP_Classifier).

## Results

The data corpus of 1000 patients was comprised of 63,301 notes, 15,651 CUI codes, and 71,987 unigrams. Classifier performance across the top neural network models for opioid misuse as well as a rule-based model and logistic regression model using CUI codes, words, and characters are displayed in Table 2.

**Table 2:** Comparison of classifiers for opioid misuse

Classifier	ROC AUC (95% CI)	F1	Precision/PPV (95% CI)	Recall/Sensitivity (95% CI)	Specificity (95% CI)	NPV (95% CI)	*P* value for model fit^*^
Rule-based	NA^a^	0.76	0.68 (0.57, 0.78)	0.87 (0.76, 0.94)	0.79 (0.71, 0.86)	0.92 (0.85, 0.96)	< 0.01
Logistic Regression CUI	0.91 (0.86, 0.95)	0.79	0.89 (0.77, 0.96)	0.71 (0.58, 0.81)	0.95 (0.90, 0.98)	0.86 (0.80, 0.91)	0.06
Logistic Regression Word	0.91 (0.86, 0.95)	0.72	0.86 (0.73, 0.94)	0.62 (0.49, 0.73)	0.95 (0.89, 0.98)	0.83 (0.76, 0.88)	< 0.01
Convolutional Neural Network CUI	0.93 (0.90, 0.97)	0.81	0.82 (0.70, 0.90)	0.79 (0.68, 0.88)	0.91 (0.85, 0.95)	0.89 (0.83, 0.94)	0.51
Convolutional Neural Network Word	0.94 (0.91, 0.98)	0.84	0.94 (0.85, 0.99)	0.75 (0.63, 0.85)	0.98 (0.93, 1.00)	0.88 (0.82, 0.93)	0.42
Convolutional Neural Network Character	0.93 (0.90, 0.97)	0.79	0.88 (0.76, 0.95)	0.72 (0.60, 0.82)	0.95 (0.89, 0.98)	0.87 (0.80, 0.92)	< 0.01
Deep Averaging Network CUI	0.83 (0.78, 0.88)	0.74	0.68 (0.57, 0.78)	0.87 (0.76, 0.94)	0.79 (0.71, 0.86)	0.92 (0.85, 0.96)	< 0.01
Deep Averaging Network Word	0.80 (0.74, 0.86)	0.49	0.74 (0.56, 0.87)	0.37 (0.25, 0.49)	0.93 (0.87, 0.97)	0.74 (0.67, 0.80)	< 0.01
Max Pooling Network CUI	0.93 (0.89, 0.96)	0.79	0.85 (0.73, 0.93)	0.74 (0.61, 0.83)	0.93 (0.87, 0.97)	0.87 (0.80, 0.92)	0.60
Max Pooling Network Word	0.91 (0.86, 0.96)	0.78	0.87 (0.76, 0.95)	0.71 (0.58, 0.81)	0.95 (0.89, 0.98)	0.86 (0.79, 0.91)	0.36
Deep Averaging + Max Pooling Network CUI	0.94 (0.91, 0.97)	0.81	0.92 (0.82, 0.98)	0.72 (0.60, 0.82)	0.97 (0.92, 0.99)	0.87 (0.80, 0.92)	< 0.01
Deep Averaging + Max Pooling Network Word	0.94 (0.91, 0.97)	0.78	0.86 (0.74, 0.94)	0.72 (0.60, 0.82)	0.94 (0.88, 0.97)	0.87 (0.80, 0.92)	0.09

Logistic regression with a combination of unigrams and bigrams; *PPV* positive predictive value, *NPV* negative predictive value, *ROC AUC* area under the curve receiver operating characteristic, *CUI* concept unique identifier, *CI* confidence interval

^*^model fit by Hosmer-Lemeshow Goodness of Fit test where *p* > 0.05 demonstrate the model fit the data well

^a^*NA* not applicable because bivariate predictions (0/1) without predicted probabilities to plot ROC AUC

**Fig. 2:**
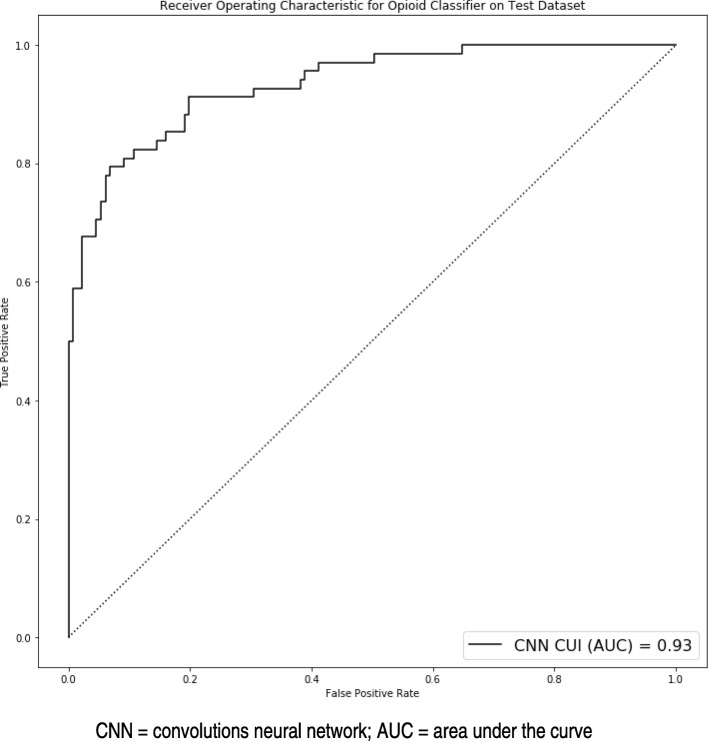
Receiver operating characteristics area under the curve for convolutional neural network model using concept unique identifiers (CUI) for classification of opioid misuse. CNN = convolutions neural network; AUC = area under the curve

The primary outcome was to optimize to the highest AUROC and the following were the top performing models: (1) CNN CUI; (2) CNN n-gram; (3) CNN character; (4) max pooling network CUI; (5) combined max pooling and deep averaging network CUI; (6) max pooling network n-gram; (7) combined max pooling and deep averaging network n-gram; (8) combined maxed pooling and deep averaging network CUI; (9) logistic regression n-gram; and (10) logistic regression CUI. In these top performing models, comparisons between the AUROC curves did not demonstrate any statistical differences (*p* > 0.05 for all comparisons). The AUROC curve for the CNN CUI model is displayed in Fig. 2**.**

**Fig. 3:**
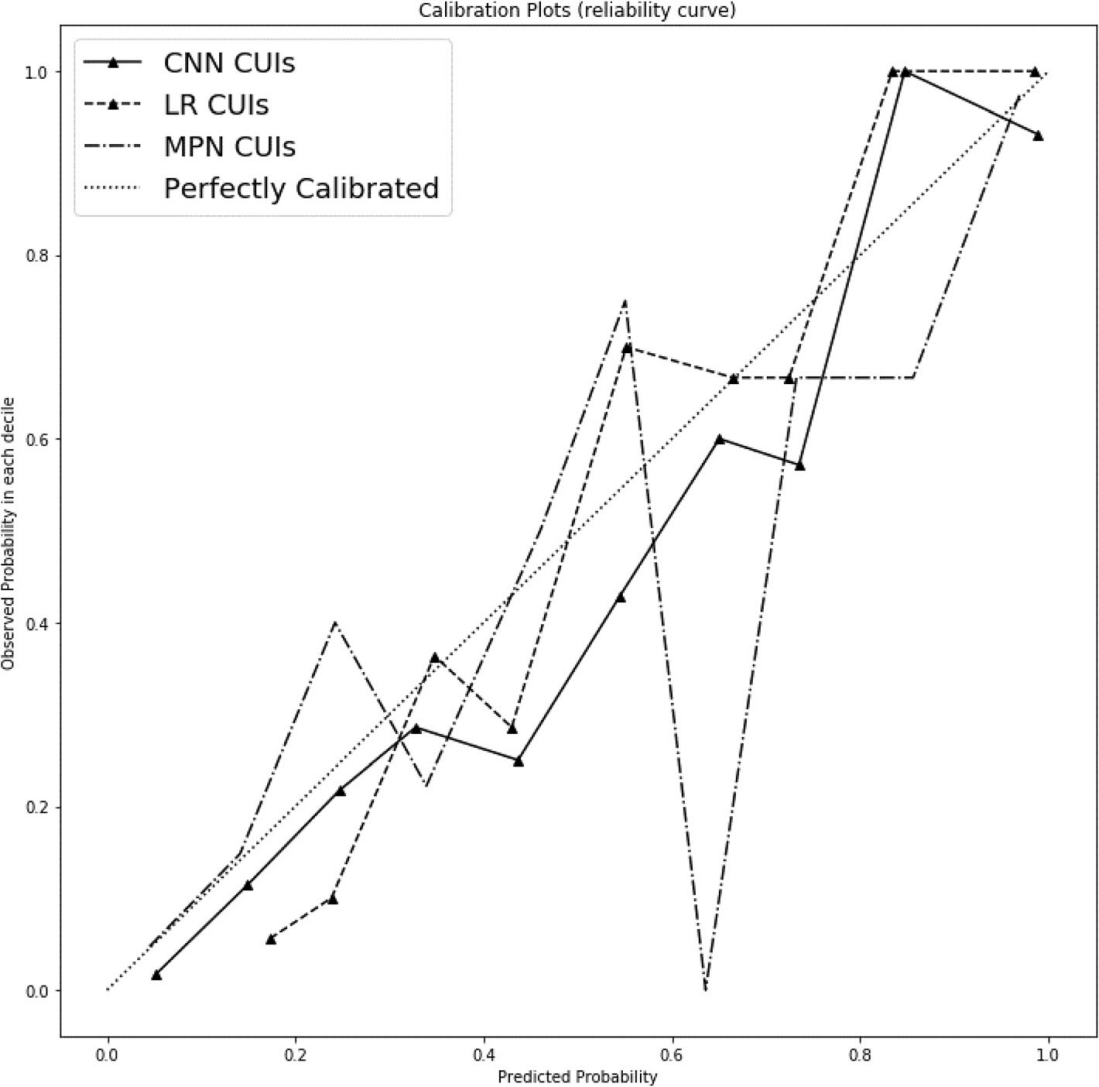
Calibration plot for top performing machine learning classifiers for opioid misuse. The diagonal line represents perfect calibration between predicted probabilities that are observed (y-axis) and predicted (x-axis). CNN = convolutions neural network; CUIs = concept unique identifiers; LR = logistic regression; MPN = max pooling network

Of these top performing models, only the CNN CUIs and n-grams, max pooling network CUIs and n-grams, and logistic regression CUIs fit the data well by Hosmer-Lemeshow test (p > 0.05). The rule-based model did not fit the data well (*p* < 0.01). The CUI codes approach for CNN, max pooling network and logistic regression visually fit the data best when plotted across deciles of predicted probabilities (Fig. 3).

In comparing n-gram to character-level and CUI-based features, similar test characteristics are noted for precision/PPV, recall/sensitivity, specificity, and NPV within the CNN and max pooling network architecture. The logistic regression CUIs had better recall/sensitivity than the logistic regression with n-grams. The CNN approach outperformed the rule-based classifier in most metrics except for recall/sensitivity and NPV. The CNN CUIs had the greatest recall/sensitivity whereas the logistic regression CUIs had the greatest precision/PPV (Table 2).

In terms of complexity, the CNN CUI model had a total number of 5,721,449 trainable parameters and the max pooling network CUI model had 4,401,257 trainable parameters. The logistic regression model selected 992 CUI code trainable parameters. L1 regularization removed zero weighted features and the final model had 21 features (Table 3). Among the selected CUI features from logistic regression, ‘heroin’ followed by ‘victim of abuse (finding)’ carried the highest beta coefficients.

**Table 3:** Concept Unique Identifiers (CUIs) for opioid misuse from logistic regression classifier and their β coefficients

CUI	Related text	β coefficients
*POSITIVE FEATURES*		
C0011892	Heroin	16.57
C0344198	Victim of abuse (finding)	12.70
C0562381	Cocaine	4.39
C0025605	Methadone	4.19
C0376196	Opiates	4.09
C0001927	Albuterol	2.40
C0728755	Dilaudid	1.73
C0029944	Drug Overdose	1.34
C0030049	Oxycodone	1.12
C0150055	Chronic pain	0.47
C0040861	Triage	0.47
C1299583	Independently able	0.19
C0022742	Knee	0.02
*NEGATIVE FEATURES*		
C0002903	Anesthesia procedures	−2.08
C0003483	Aorta	−1.51
C0006826	Malignant Neoplasms	−1.50
C1272883	Injection	−1.36
C0006434	Burn injury	−0.71
C0020538	Hypertensive disease	−0.42
C0021641	Insulin	−0.09
C0004604	Back Pain	−0.01

## Discussion

CUI code inputs to a CNN, max pooling network, and logistic regression model had the optimal performance for an opioid misuse classifier with AUROCs greater than 0.90. CUI codes are a standardized and automated feature representation that require no domain expertise and can be extracted using off-the-shelf software. Our main finding was that PHI-free models were similar to or outperformed models containing PHI. The PHI-free neural network models (CNN and max pooling network with CUI codes) were the top calibrated models with the best discrimination and are available to health systems interested in deploying these black box models with the assurance that they are compliant with the Health Insurance Portability and Accountability Act (HIPAA). The PHI-free, trained opioid misuse computable phenotypes are available on GitHub for other researchers and health systems to apply (https://github.com/AfsharJoyceInfoLab/OpioidNLP_Classifier).

An estimated 80% of all data in EHRs reside in clinical notes [22, 23] and are a rich source of data, but their unstructured format makes them complex and difficult to de-identify. Recent methods for identification of the clinical notes have achieved above 90% in accuracy and F1 scores [24–26]. However, this does not constitute as fully PHI-free data and poses a barrier for health systems to share data legally. The legal requirements from HIPAA were recently highlighted in a federal class-action lawsuit making a claim that notes in the EHR of a major health system did not meet the requirements for a fully de-identified dataset [27]. Clinical notes lack common structural frameworks, contain many grammatical and spelling errors, lexical variation, and are often semantically ambiguous making de-identification difficult [28]. Methods in NLP, including concept mapping to CUI codes to produce a standard medical vocabulary, are a more effective and efficient approach for automatic semantic analyses of clinical notes [29]. We have previously shown success in using a CUI code-based approach for a NLP classifier to identify alcohol misuse and respiratory failure [30, 31].

Given the complexities of behavioral conditions like opioid misuse, very little data are available demonstrating useful computable phenotypes from CUI codes or character-based approaches. A systematic review of computable phenotypes for opioid misuse revealed the data used in many published algorithms are not routinely available in the EHR, use PHI-laden machine learning models, or rely solely on diagnostic billing codes [11]. To date, the best performing algorithms depend on pharmacy claims data which are not available in EHRs; therefore, are impractical to implement for providers and hospitals [32–34]. Text classification from clinical notes has demonstrated good test characteristics but have been focused in certain subtypes or specific cohorts of patients with opioid misuse and contain PHI [4, 35]. Our study using a CUI-based machine learning approach for predicting all types of opioid misuse provides a PHI-free solution and also accounts for lexical variations and semantic ambiguities. In this approach, discovery is not limited by domain knowledge or expertise, and other entity mentions outside the opioid domain may prove predictive.

Identification of opioid misuse incorporates a continuum of individuals who may occasionally use opioids for non-medical purposes to opioid use disorders, and these individuals commonly have co-occurring mental health conditions and polysubstance use [36–38]. In examining the CUI codes selected from logistic regression, this becomes apparent as clinically relevant concepts that are not explicit mentions of opioid drugs such as pain conditions, victims of abuse, and adverse events of opioid misuse are identified [39]. Also noteworthy is the logistic regression classifier identified negative features such as malignant neoplasms and acute pain conditions that are not relevant to opioid misuse. The character-based approaches for opioid misuse also proved to be useful and have similar discrimination to the logistic regression n-gram model but face validity is not apparent in the more complex, neural network architecture.

Our group has previously shown advantages to opioid misuse phenotyping using transfer learning, but this paradigm is more beneficial for tasks with small sample sizes of training data [40]. In addition, transfer learning further obscures the feature extraction from more conventional neural architectures, which may be less appealing to healthcare providers. Our study utilized a training set with adequate size to achieve AUROCs above 0.90 and obviated any need for transfer learning. A future direction in this field of research involves extending pre-trained models like the Bidirectional Encoder Representations from Transformers (BERT) to very long sequences of text (e.g., thousands of tokens).

Potential limitations of our approach include a data corpus from a single hospital that may not represent the practice variations across hospitals. Further, social and behavioral determinants of health are typically limited in the EHR or not available at all [41]; however, substance use is a routinely captured data metric during intake in notes by providers, which is why we focused only on notes. The CUI approach did not prove to have appreciable differences in performance from an n-gram approach; however, a lot of the medical vocabulary may be lost during concept mapping to CUIs. This may prove to be a limitation at other health systems and potentially lead to high variance in extracted medical terms and, ultimately, performance of the classifier. For example, while some non-standard terms such as “dope” and “speedball” have CUIs, these terms may be unique to the catchment of the health system with other slang used in other regions of the US. Further misspellings of heroin and opiate/opioid, undocumented sentence structure, and other lexical variations may further contribute to the variability in mapped CUI features. Lastly, the computing resources needed to produce CUI codes and process large amounts of notes may not be readily available at other hospitals, but we have previously published a pipeline architecture for clinical data warehouses to help overcome this barrier [42].

## Conclusion

PHI-free approaches for building computable phenotypes from clinical notes are needed for better surveillance and case-identification. Opioid misuse is a complex behavioral condition that requires information contained in the clinical notes, and machine learning approaches for text classification are a viable solution for case-identification. Our computable phenotypes for opioid misuse may prove useful to health systems for more accurate identification and surveillance of hospitalized patients without the risk of leaking any PHI.

## Data Availability

The trained models generated from this study are available on the corresponding author’s GitHub repository (https://github.com/AfsharJoyceInfoLab/OpioidNLP_Classifier). The original patient data used for model training are only available from Loyola University Medical Center and Loyola University, where the data were extracted from the hospital’s electronic health record (EHR) and contains patients’ protected health information (PHI). These data can only be accessed for researchers who meet the legal and regulatory criteria from the institution for access to confidential data, including a data usage agreement and IRB approval with both Loyola University Chicago and Loyola University Medical Center. Requests for data may be initiated with the corresponding author (majid.afshar@lumc.edu).

## Abbreviations

PHI: Protected health information
CUI: Concept unique identifier
NLP: Natural language processing
HER: Electronic health record
ICD: International classification of disease
cTAKES: Clinical text and knowledge extraction system
UMLS: Unified medical language system
ROC AUC: Receiver operating characteristic area under the curve
PPV: Positive predictive value
NPV: Negative predictive value
CNN: Convolutional neural network
CDW: Clinical data warehouse
LUMC: Loyola University Medical Center
